# Characteristics of intracerebral haemorrhage associated with COVID-19: a systematic review and pooled analysis of individual patient and aggregate data

**DOI:** 10.1007/s00415-021-10425-9

**Published:** 2021-02-05

**Authors:** R. Beyrouti, J. G. Best, A. Chandratheva, R. J. Perry, D. J. Werring

**Affiliations:** 1 Comprehensive Stroke Service, National Hospital for Neurology and Neurosurgery, University College Hospitals NHS Foundation Trust, Queen Square, London, WC1N 3BG UK; 2 Stroke Research Centre, Department of Brain Repair and Rehabilitation, UCL Queen Square Institute of Neurology, First Floor, Russell Square House, 10-12 Russell Square, London, WC1B 5EH UK

**Keywords:** COVID-19, Intracerebral haemorrhage, Stroke, Anticoagulation

## Abstract

**Background and purpose:**

There are very few studies of the characteristics and causes of ICH in COVID-19, yet such data are essential to guide clinicians in clinical management, including challenging anticoagulation decisions. We aimed to describe the characteristics of spontaneous symptomatic intracerebral haemorrhage (ICH) associated with COVID-19.

**Methods:**

We systematically searched PubMed, Embase and the Cochrane Central Database for data from patients with SARS-CoV-2 detected prior to or within 7 days after symptomatic ICH. We did a pooled analysis of individual patient data, then combined data from this pooled analysis with aggregate-level data.

**Results:**

We included data from 139 patients (98 with individual data and 41 with aggregate-level data). In our pooled individual data analysis, the median age (IQR) was 60 (53–67) years and 64% (95% CI 54–73.7%) were male; 79% (95% CI 70.0–86.9%) had critically severe COVID-19. The pooled prevalence of lobar ICH was 67% (95% CI 56.3–76.0%), and of multifocal ICH was 36% (95% CI 26.4–47.0%). 71% (95% CI 61.0–80.4%) of patients were treated with anticoagulation (58% (95% CI 48–67.8%) therapeutic). The median NIHSS was 28 (IQR 15–28); mortality was 54% (95% CI 43.7–64.2%). Our combined analysis of individual and aggregate data showed similar findings. The pooled incidence of ICH across 12 cohort studies of inpatients with COVID-19 (*n* = 63,390) was 0.38% (95% CI 0.22–0.58%).

**Conclusions:**

Our data suggest that ICH associated with COVID-19 has different characteristics compared to ICH not associated with COVID-19, including frequent lobar location and multifocality, a high rate of anticoagulation, and high mortality. These observations suggest different underlying mechanisms of ICH in COVID-19 with potential implications for clinical treatment and trials.

**Supplementary Information:**

The online version contains supplementary material available at 10.1007/s00415-021-10425-9.

## Introduction

Coronavirus Disease 2019 (COVID-19) can be complicated by a coagulopathy with a high risk of serious and often life-threatening thromboembolic events including venous thromboembolism [1, 2] and ischaemic stroke [3]. This has led to the widespread use of anticoagulants including heparins, often guided by D-dimer levels as evidence for the degree of hypercoagulability [4]. While anticoagulants appear to improve outcome in severe COVID-19 [5], they also have the potential to increase the risk of serious intracranial bleeding, particularly intracerebral haemorrhage (ICH).

There are a few data available on the clinical features of ICH in people with COVID-19 [6, 7]. Previous reports did not allow for clear conclusions due to small sample sizes. Information about the characteristics and causes of ICH in COVID-19 is essential to guide clinicians in optimising clinical management, including making difficult anticoagulation decisions. We, therefore, undertook a systematic review and pooled analysis of all available studies reporting individual or aggregate-level patient data on ICH in the context of COVID-19.

## Methods

### Search strategy and study selection

We carried out a systematic literature search from PubMed, Embase and Cochrane Central Database, according to Preferred Reporting Items for Systematic Reviews and Meta-Analyses (PRISMA) guidelines [8], with the following search terms ((“stroke” OR “cerebrovascular” OR “intracranial” OR “intracerebral” OR “intraparenchymal”) AND (“haemorrh*” OR “hemorrh*”)) AND (“COVID-19” OR “coronavirus” OR “corona virus” OR “Coronavirus” OR “2019-nCoV” OR “SARS-CoV” OR “Severe Acute Respiratory Syndrome”). We limited the results to 2019–2020 to exclude papers referring to other coronaviruses. We did not limit the search by language. We used EndNote software to run auto de-duplication. After removal of duplicates, two reviewers (RB and JB) performed an initial search and screening for relevant articles through title and abstract; discrepancies were resolved by consensus, and where necessary with a third senior reviewer (DJW). The potential full texts were evaluated by applying the inclusion and exclusion criteria given below. The literature search was finalized on December 12, 2020.

### Inclusion and exclusion criteria

We included all studies that reported patients confirmed to have severe acute respiratory syndrome coronavirus 2 (SARS-CoV-2) infection according to the WHO interim guideline [9] (i.e. a nasopharyngeal or oropharyngeal swab positive for SARS-CoV-2 on reverse transcriptase polymerase chain reaction (RT-PCR), or a blood sample positive for SARS-CoV-2 antibodies) with SARS-CoV-2 detected either prior to, at the time of, or up to 7 days post, spontaneous (nontraumatic) symptomatic ICH. We excluded studies reporting other types of intracranial haemorrhage including microhaemorrhages, ischaemic infarcts with haemorrhagic transformation, subdural hematoma, subarachnoid haemorrhage and haemorrhagic leukoencephalopathy. Review articles, editorials, opinion papers and guidelines were excluded. For the pooled individual patient data analysis, we included papers that presented individual-level data on any of our variables of interest. Papers presenting aggregate-level data only were subsequently included in a pooled analysis of both individual patient and aggregate data.

### Data extraction

The following variables were extracted from the studies: demographics (age, gender); vascular risk factors; ICH characteristics (location, cause); stroke severity (National Institutes of Health Stroke Scale (NIHSS) score); anticoagulant treatment (prophylactic or therapeutic) at the time of ICH; COVID-19 severity and clinical outcome; coagulation and inflammation markers (D-dimers and fibrinogen, APPT, PT, INR, platelets, CRP, ferritin). We classified ICH from reported information according to location as lobar or non-lobar (i.e. deep or infratentorial, defined as ICH in the basal ganglia, thalamus, brainstem or the cerebellum). We then classified ICH aetiology using all information available by predefined criteria as follows: hypertensive arteriopathy (deep or infratentorial haemorrhage with known hypertension before the ICH); cerebral amyloid angiopathy (defined as ≥ 1 lobar, cortical, or cortico-subcortical haemorrhage and age ≥ 55 with no other identified cause); structural macrovascular lesions (aneurysm, pseudoaneurysm, cavernous malformation); coagulopathy (defined as PT ≥ 15 and/or INR ≥ 1.2 and/or APTT ≥ 45 and/or platelets ≤ 100,000); other identified causes (cerebral venous thrombosis, Moya Moya angiopathy); or undetermined.

To estimate the pooled incidence of ICH in patients with COVID-19, we extracted data on the number of hospitalised patients with COVID-19 and the number documented to have ICH in the same time period.

### Statistical analysis

A pooled analysis was performed on all of the individual patient data found in the published literature, to estimate means, medians, standard deviations, minimum and maximum values. We then combined the aggregate data from these individual patients with other studies reporting aggregate data according to the two-stage method [10], using a random-effects model. We compared the characteristics of patients who were and were not receiving anticoagulation at the time of their ICH using Fisher’s exact test. Statistical analyses were performed using Microsoft Office Excel version 365 16.0 and Stata 16.

## Results

### Literature search and screening

**Fig. 1:**
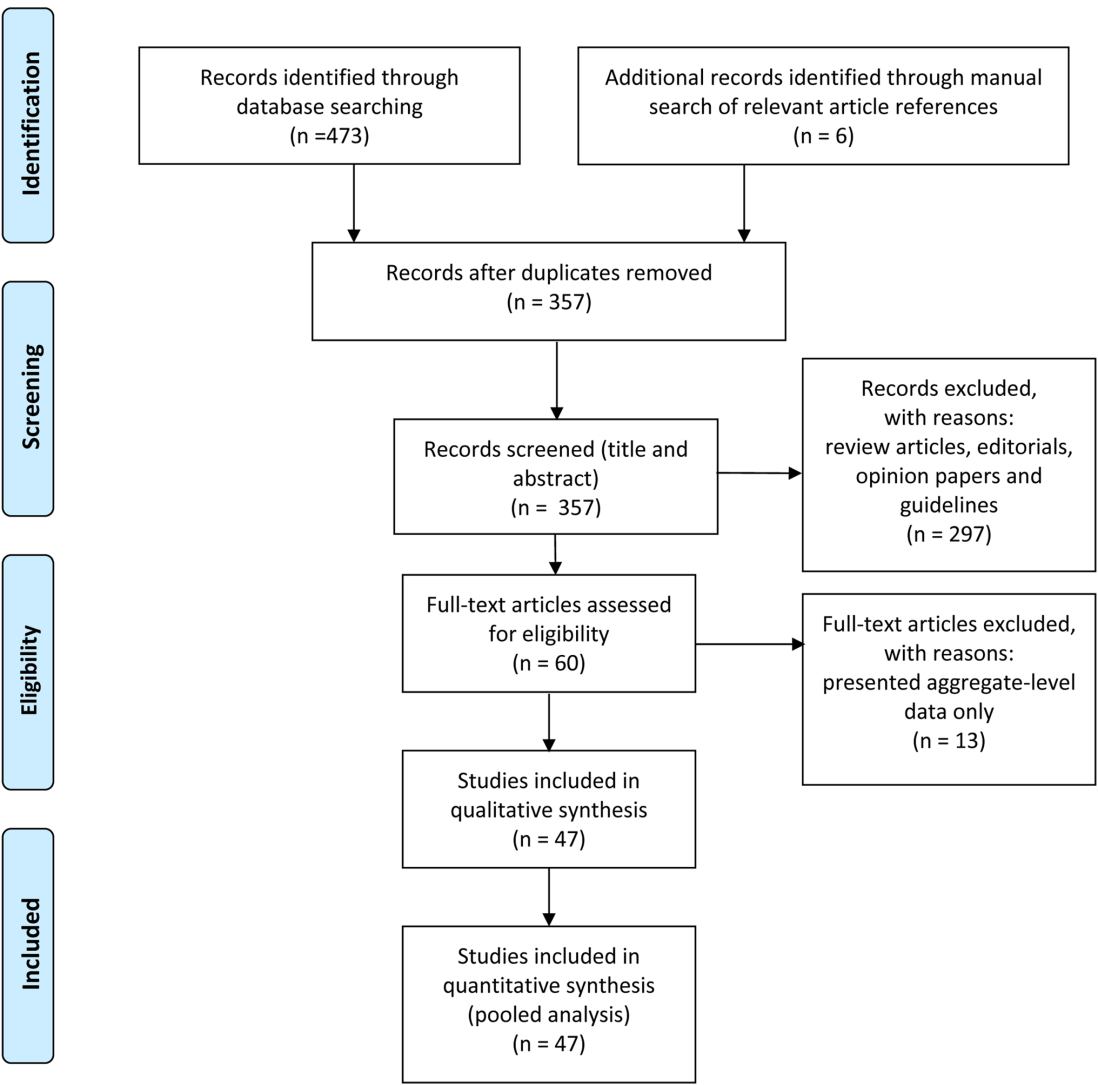
Flow diagram of the literature search and selection process in the pooled individual patient data analysis

The flow diagram (Fig. 1) shows the detailed literature search steps and data selection for our individual patient data pooled analysis. The database searches identified a total of 473 potentially relevant articles. Six further articles were added through a manual search of relevant article references. After the exclusion of duplicate references, 357 articles were considered for the pooled individual data analysis. 297 studies were excluded after screening the title and abstract because they were review articles, editorials, opinion papers and guidelines. A total of 47 studies with individual-level data on our variables of interest qualified for inclusion, identifying 98 reported patients with COVID-19 and ICH. A further 13 studies reported only aggregate-level data on patients with COVID-19-associated intracranial haemorrhage not already included in our individual patient data meta-analysis. These contained 190 cases of intracranial haemorrhage, of which 99 were intracerebral. Five studies including 41 patients with ICH reported ICH-specific data (see Table 1) and were eligible for our two-stage pooled analysis.

**Table 1:** Aggregate data summary

Paper	Setting	Number of ICH cases	Data available
Trifan 2020 [6]	Multihospital case series, US	16	Sex, ICH topography, anticoagulation
Dogra 2020 [20]	Multihospital case series, US	5	Age, sex, risk factors, anticoagulation
Nawabi 2020 [37]	Multihospital case series, Europe	6	ICH topography
Rothstein 2020 [38]	Multihospital case series, US; three patients on ECMO	5	ICH topography, anticoagulation
Altschul 2020 [39]	Multihospital case series, US	9	Age, sex, risk factors, anticoagulation

### Pooled individual patient data analysis

#### Patient demographics and intracerebral haemorrhage characteristics

The characteristics of the patients included in our pooled individual patient data analysis (*n* = 98) are shown in Table 2; 79% (95% CI 70.0–86.9%) had critically severe COVID-19. Detailed data from all individual patients included are shown in Supplementary Table 2. The median age was 60 years (IQR 53–67); 64% were men. The proportions of patients with vascular risk factors were: hypertension in 53%, diabetes in 29%, dyslipidaemia in 16%, high BMI in 11% and history of smoking in 11% (Table 2); 20% had no history of vascular risk factors.

**Table 2:** Characteristics of patients included in individual patient data pooled analysis

Demographics (*n* = 98)	
Age - Median (IQR)	60 (53–67)
Male % (95% CI)	64% (54–73.7%)
Vascular risk factors: (*n* = 91)	*N* (%, 95% CI)
Hypertension	48 (53%, 42.0–63.3%)
Diabetes	26 (29%, 19.6–39.0%)
Dyslipidaemia	15 (16%, 9.5–25.7%)
Smoking	10 (11%, 5.4–19.3%)
High body mass index	10 (11%, 5.4–19.3%)
Atrial fibrillation	3 (3%, 0.7–9.3%)
Ischemic heart disease	3 (3%, 0.7–9.3%)
Alcohol	2 (2%, 0.3–7.7%)
No previous medical history	18 (20%, 12.1–29.5%)
Intracerebral haemorrhage characteristics	*N* (%, 95% CI)
Location (*n* = 96)	
Lobar	64 (67%, 56.3–76.0%)
Non-lobar (deep or infratentorial)	28 (29%, 20.3–39.3%)
Mixed (lobar and non-lobar)	4 (4%, 1.15–10.3%)
Multifocality (*n* = 91)	
Multifocal	33 (36%, 26.4–47.0%)
Focal	58 (64%, 53.0–73.6%)
Aetiology (*n* = 98)	
Coagulopathy	50 (51%, 40.7–61.3%)
Hypertensive arteriopathy	11 (11%, 5.7–19.2%)
Cerebral amyloid angiopathy	1 (1%, 0.0–5.6%)
Macrovascular causes (*)	3 (3%, 0.6–8.7%)
Cerebral venous thrombosis	1 (1%, 0.0–5.6%)
Moya-Moya disease	1 (1%, 0.0–5.6%)
Undetermined	31 (32%, 22.6–41.8%)
Anticoagulation (*n* = 91)	*N* (%, 95% CI)
Therapeutic unfractionated heparin	36 (40%, 29.5–50.4%)
Therapeutic low molecular weight heparin	14 (15%, 8.7–24.5%)
Prophylactic low molecular weight heparin	12 (13%, 7.0–21.9%)
Warfarin	3 (3%, 0.7–9.3%)
No anticoagulation	26 (29%, 19.6–39.0%)
COVID-19	*N* (%, 95% CI)
Prior symptoms (*n* = 92)	
Prior COVID-19 symptoms	71 (77%, 67.3–85.3%)
No prior COVID-19 symptoms	21 (23%, 14.7–32.8%)
Days since COVID-19 symptoms (*n* = 68)	
Median, IQR	15 (5–20)
Severity (*n* = 97)	
Critical	77 (79%, 70.0–86.9%)
Severe	4 (4%, 1.1–10.2%)
Moderate	9 (9%, 4.3–16.9%)
Mild	2 (2%, 0.3–7.3%)
Asymptomatic	5 (5%, 1.7–11.6%)
ECMO (*n* = 98)	20 (21%, 12.9–29.8%)
Outcome (*n* = 98)	
Deceased	53 (54%, 43.7–64.2%)
Critically ill	18 (18%, 11.3–27.5%)
Discharged to rehabilitation unit or home	27 (28%, 19.0–37.5%)
Coagulability markers	Median, IQR
D-Dimer (μg/L) (*n* = 64)	3387 (1745–5670)
Fibrinogen (g/L): (*n* = 42)	5 (3.6–6.5)
aPTT (s) (*n* = 36)	32 (25–59.5)
PTT (s) (*n* = 21)	64 (41.6–89.3)
INR (*n* = 45)	1.2 (1.1–1.5)
Prothrombin time (s) (*n* = 28)	12.8 (11.7–15.7)
Platelet count (/ mm3) (*n* = 72)	211 (143, 288.5 k)
CRP (mg/L) (*n* = 57)	74 (13–130)
Serum ferritin (μg/L) (*n* = 20)	1554 (983–2289)

*Macrovascular causes (aneurysm, arteriovenous malformation, cavernous malformation)

*ECMO* extracorporeal membrane oxygenation, *aPTT* activated partial thromboplastin time, *PTT* prothrombin time, *INR* international normalised ratio, *CRP* C-reactive protein

ICH was frequently lobar (67%), multifocal (36%) and severe, with a median NIHSS score of 28 (IQR 15–28; available in 18 patients). Non-lobar (deep or infratentorial) ICHs were less common (29%), while mixed (lobar and non-lobar) ICHs were reported in only 4% of cases. Coagulopathy was the most common reported cause of ICH (50/98; 51%), nearly always attributed to therapeutic anticoagulation (48/50; 96%).

65/91 (71%) patients were receiving anticoagulation at the time of ICH, which was therapeutic in 53/91 (58%): therapeutic unfractionated heparin (UFH) in 36 (40%); therapeutic low molecular weight heparin (LMWH) in 14 (15%); and warfarin in 3 (3%). Prophylactic LMWH was used in 12/91 (13%). The indication for anticoagulation was extracorporeal membrane oxygenation (ECMO) in 20, concurrent thromboembolic events (pulmonary embolism and deep venous thrombosis) in 5, atrial fibrillation in 3, mechanical cardiac valve replacement in one patient and occlusive femoral artery thrombus in one. 35 patients were started on either therapeutic or prophylactic anticoagulation for suspected COVID-19 hypercoagulability based on local protocols.

The characteristics of patients with known anticoagulation status are shown in Table 3. 26/91 (29%, 95% CI 20–39%) patients were not receiving any anticoagulation at the time of ICH. Of these, 12/26 (46%, 95% CI 27–67%) were lobar, 2/23 (9%, 95% CI 1–28%) were multifocal, 7/26(27%, 95% CI 12–48%) were attributed to hypertensive arteriopathy, and 1/26(4%, 95% CI 0–20%) was associated with coagulopathy. The proportions of patients with lobar ICH and multifocal ICH were lower in those who were not anticoagulated compared to those who were anticoagulated (12/26 (46%, 27–67%) vs 51/63 (81%, 69–90%), *p* = 0.002; and (2/23 (9%, 1–28%) vs 31/63 (49%, 36–62%), *p* < 0.001, respectively). The proportion of ICH attributed to coagulopathy was 1/26 (4%, 0–20%) in patients who were not anticoagulated compared to 49/65 (75%, 63–85%) in those who were (p < 0.001). The proportion of ICH attributed to hypertensive arteriopathy was higher in patients who were not anticoagulated compared to those who were (7/26 (27%, 12–48%) vs 0/65 (0%, 0–5%), *p* < 0.001).

**Table 3:** ICH characteristics in non-anticoagulated versus anticoagulated patients

	Non-anticoagulated (%, 95% CI)	Anticoagulated (%, 95% CI)
Patients	26/91 (29%, 20–39%)	65/91 (71%, 61–80%)
Location	Available in 26/26	Available in 63/65
Lobar	12/26 (46%, 27–67%)	51/63 (81%, 69–90%)
Non-lobar (deep or infratentorial)	14/26 (54%, 33–73%)	8/63 (13%, 6–24%)
Mixed (lobar and non-lobar)	0/26 (0%, 0–11%)	4/63 (6%, 2–15%)
Focality	Available in 23/26	Available in 63/65
Multifocal	2/23 (9%, 1–28%)	31/63 (49%, 36–62%)
Focal	21/23 (91%, 72–99%)	32/63 (51%, 38–64%)
Mechanism	Available in 26/26	Available in 65/65
Coagulopathy	1/26 (4%, 0–20%)	49/65 (75%, 63–85%)
Hypertensive arteriopathy	7/26 (27%, 12–48%)	0/65 (0%, 0–5%)
CAA	1/26 (4%, 0–20%)	0/65 (0%, 0–5%)
CVT	0/26 (0%, 0–11%)	1/65 (2%, 0–8%)
Moya-Moya disease	1/26 (4%, 0–20%)	0/65 (0%, 0–5%)
Macrovascular causes	2/26 (8%, 1–25%)	1/65 (2%, 0–8%)
Undetermined	14/26 (54%, 33–73%)	14/65 (22%, 12–33%)

Coagulability markers are summarized in Table 2. D-dimers were reported in 64/98 (65%) patients; they were elevated (> 500 μg/L) in 94% of cases with a median of 3387 μg/L (IQR 1745–5670).

#### Coronavirus disease severity

The time of onset of COVID-19 symptoms (where present) in relation to ICH was reported for 92/98 (94%) patients. Of these, 71 (77%) developed symptoms before ICH with a median interval of 15 days (IQR 5–20), while 21 (23%) had no symptoms at the time of ICH. 77 (79%) were critically ill (developed respiratory failure requiring mechanical ventilation or septic shock or other organ dysfunction or failure that required intensive care [11]) including 20 (21%) who received extracorporeal membrane oxygenation (ECMO) treatment. A further four patients (4%) had severe disease (with one of the following criteria: tachypnoea ≥ 30 breaths per min; or O2 sat ≤ 93% at rest; or PaO2/FiO2 ratio < 300 mmHg [11]), 9 (9%) had moderate disease activity (with fever, respiratory tract symptoms and pneumonia on imaging [11]), 2 (2%) had mild symptoms and no pneumonia on imaging and 5 (5%) were asymptomatic.

#### Outcome

The mortality was 53/98 (54%); 18/98 (18%) remained critically ill and 27/98 (28%) were discharged home or to a rehabilitation unit.

### Pooled analysis of individual patient and aggregate data

**Fig. 2:**
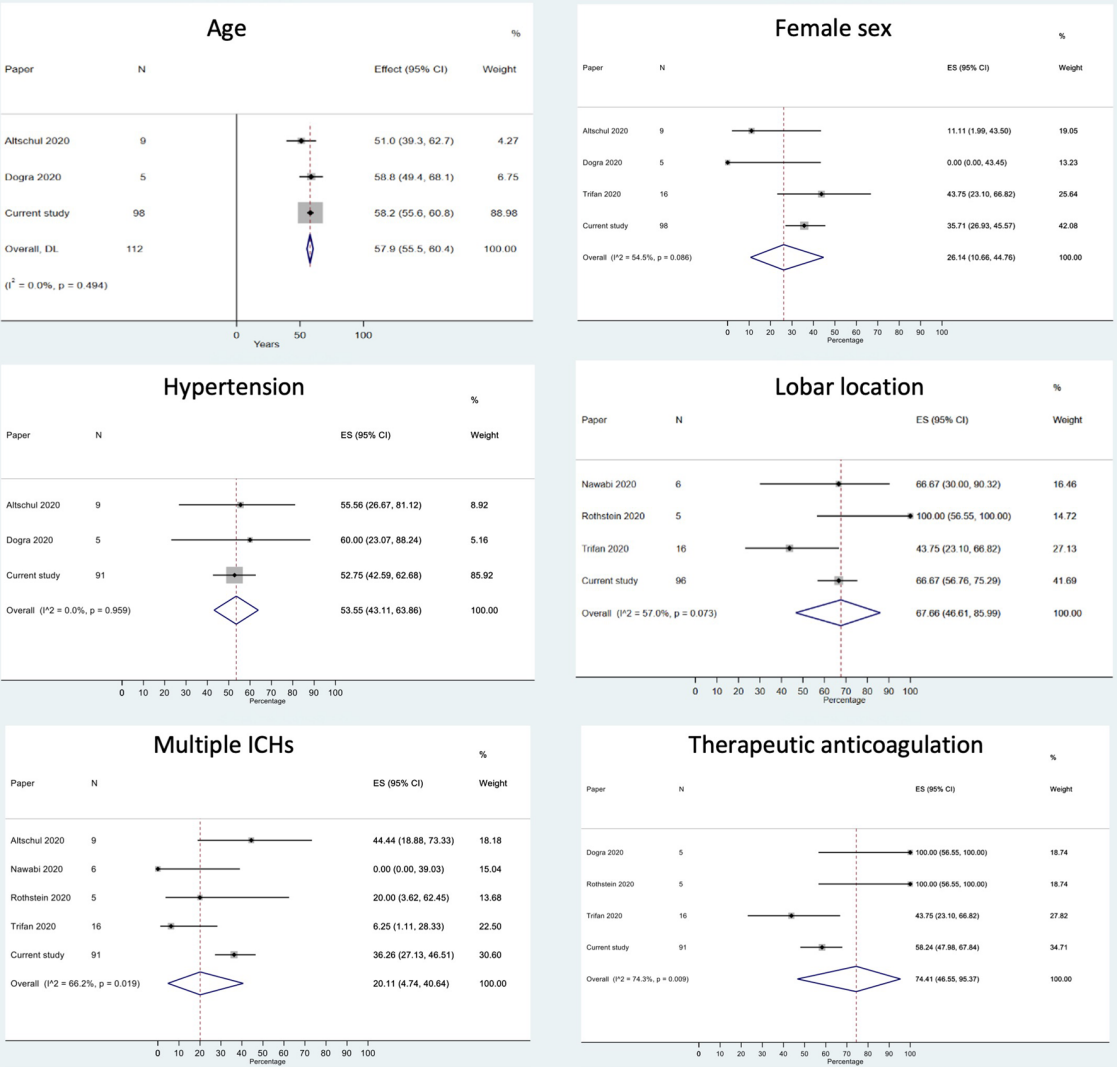
ICH characteristics in our two-stage pooled analysis of individual patient and aggregate data

Our two-stage pooled analysis included the pooled individual patient data (Table 2, *n* = 98) and ICH-specific aggregate-level data (Table 1, *n* = 41). The results for key variables are shown in Fig. 2. The mean age was 57.9 years (95% CI 55.5–60.4 years). The estimated proportion of patients of the male sex was 73.9% (95% CI 66.0–81.2%). 53.6% (95% CI 43.1–63.9%) were hypertensive, and 74.4% (95% CI 46.6–95.4%) were prescribed therapeutic-dose anticoagulation at the time of ICH. 67.7% (95% CI 46.6–86.0) of ICH were lobar, and 20.1% (95% CI 4.7–40.6%) were multifocal.

### Incidence of ICH in COVID-19

**Fig. 3:**
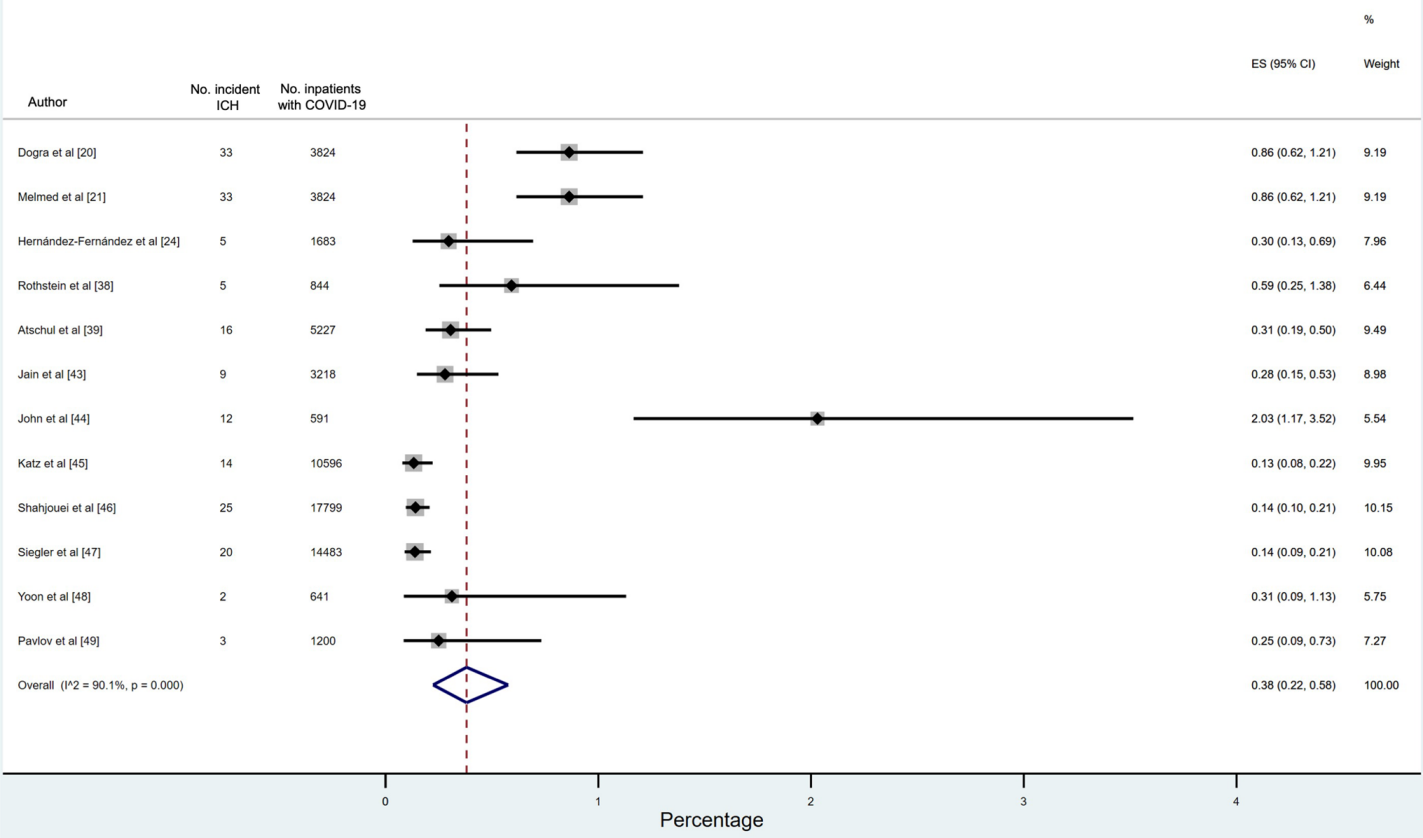
Incidence of ICH in inpatients with COVID-19

Twelve cohort studies (*n* = 63,390 patients) reported data on the incidence of ICH in COVID-19 inpatients, with the incidence ranging from 0.13 to 2.03%. The pooled incidence of ICH across these studies was 0.38% (95% CI 0.22–0.58%) (Fig. 3).

## Discussion

Spontaneous (non-traumatic) ICH in patients with COVID-19 appears to have distinct characteristics that differ from those previously reported for ICH not associated with COVID-19. Based on our pooled individual patient data analysis, in comparison to non-COVID-19 populations [12, 13], ICH associated with COVID-19 seems to affect a younger (median age 60), more frequently male (64%) and less often hypertensive (53%) population, and appears to be more often lobar, multifocal, and associated with the use of anticoagulant drugs. Our pooled analysis of individual and aggregate data showed similar findings. These results are potentially relevant for understanding underlying mechanisms, and for the management of patients with COVID-19, including the use of anticoagulants to treat or prevent thromboembolic complications. We found a low incidence of ICH in patients with COVID-19 (0.38%), but the high mortality rate (54%) suggests that ICH is among the most severe neurological complications of COVID-19. Furthermore, our data suggest that the majority of ICH occur in the context of critically severe COVID-19.

The high prevalence of lobar ICH (in 67% of patients in our pooled individual patient analysis, and 67.7% in the pooled analysis of individual and aggregate data) is striking, and consistent with the pattern recently described in a systematic review of neuroimaging features of COVID-19 [14]. In non-COVID-19 ICH populations, lobar ICH typically accounts for only 32–38% of cases [15–17]. In non-COVID-19 cohorts, lobar ICHs are mainly due to either macrovascular causes (arteriovenous malformations or aneurysms), hyaline arteriolosclerosis or cerebral amyloid angiopathy [18].

Another important finding was that ICH in COVID-19 was multifocal in 36% of patients in our pooled individual patient analysis and 20.6% in our two-stage pooled analysis; this is much more frequent than in typical ICH cohorts in which ICH are multiple in only about 6% of cases [19]. Interestingly this previous study also found that multiple ICHs were more often lobar and associated with systemic coagulopathy [19]. We found hypertension in only 53% of cases, also in keeping with the data in multiple non-COVID-19 ICH [19]. In COVID-19 patients not on anticoagulants, the proportion of multifocal ICH was much lower (9%) than in our full cohort (49%), and similar to the general rate of ICH multifocality in non-COVID-19 ICH populations, suggesting that anticoagulation is an important precipitating factor for multifocal ICH in patients with COVID-19.

The unusual characteristics of ICH associated with COVID-19 raise the possibility that the underlying mechanisms might be different from those of spontaneous ICH in patients without COVID-19. In our pooled individual and aggregate data analysis, 74.4% of ICH were associated with therapeutic anticoagulation, which may have been an important contributory factor. It is also possible that ICH occurs in more severe COVID-19, in which anticoagulation is more likely to be given (including for ECMO). Our findings are consistent with a recent study reporting 33 patients with COVID-19 and intracranial bleeding (primary ICH in 5 patients and haemorrhagic transformation of an infarct in most of the rest) and a high rate of anticoagulation (66.7% therapeutic anticoagulation and 9.1% prophylactic anticoagulation) [20]. In another study, therapeutic anticoagulation use (compared with not using anticoagulation) in COVID-19 patients was associated with a five-fold increased risk of ICH [21]. By contrast, in previous mechanistic classifications of non-COVID-19 ICH, anticoagulation accounted for only 10–15% of cases [22]. Interestingly, only 4% of ICHs were associated with coagulopathy in non-anticoagulated patients, supporting the hypothesis that COVID-19-associated ICH can result from vessel wall pathology rather than requiring the presence of impaired clotting associated with a critical illness, anticoagulation therapy, or both.

D-dimer levels were high in most patients (median (IQR) 3387 μg/L (IQR 1745–5670), surpassing the threshold identified as a predictor of in-hospital mortality [23] and consistent with a recent study in COVID-19 associated acute stroke that found elevated D-Dimers in all patients with haemorrhagic stroke (mean of 3,387 μg/L), which was lower than the levels observed in ischaemic stroke (mean 7148 μg/L) [24]. Arterial and venous thromboembolic events, including ischaemic strokes, have been widely associated with severe coronavirus disease [25], while cerebrovascular disease is associated with increased disease severity in patients with COVID-19 [26]. Similarly, we found that the majority of ICH (79%) occurred in patients with critically severe COVID-19. These findings, together with the findings from the current study, emphasise the challenging combination of thrombotic and haemorrhagic complications, particularly in severe COVID-19 [27].

The pathogenesis of intracerebral haemorrhage in COVID-19 patients is likely to be complex. One report describing 41 cases of COVID-19 indicated that prolonged prothrombin time, elevated D-dimer, and severe platelet reduction occur in critical COVID-19 patients [28], all of which are associated with increased propensity for haemorrhagic complications. Although anticoagulation was common in our study, it is not considered to be a sufficient or necessary cause for ICH but is hypothesised to aggravate haemorrhage from vessels prone to bleeding due to other pathologies. SARS-CoV-2 has both direct and indirect effects on the cerebral vasculature that could increase the risk of haemorrhage through various potential mechanisms [29]. First, it has been widely reported that SARS-CoV-2 binds to the angiotensin-converting enzyme 2 (ACE2) receptors largely present in the brain vessels [30] which could cause endothelial injury and blood–brain barrier (BBB) disruption [31]. Endothelial cell infection and endotheliitis have been demonstrated in COVID-19 via histological identification of viral elements within endothelial cells and an accumulation of inflammatory cells and apoptosis [32]. A diffuse bleeding-prone endotheliitis, perhaps aggravated by the use of therapeutic anticoagulation, could thus be a contributory factor in explaining our findings. Other studies in COVID-19 have found evidence of a haemorrhagic leukoencephalopathy with multiple cerebral microbleeds, in keeping with the presence of a diffuse haemorrhage-prone vasculopathy [33]. Other accumulating evidence suggests that SARS-CoV-2 may be associated with a cytokine storm syndrome and excessive oxidative stress [34], which could disrupt endothelial function, causing blood–brain-barrier breakdown and ICH [35]. Finally, disruption of the renin-angiotensin system (RAS) may also play a role in COVID-19-mediated ICH; down-regulation of endothelial ACE2 receptors can cause dys-autoregulation of cerebral blood flow [36].

The majority of patients with COVID-19-associated ICH (77%) presented with COVID-19 symptoms prior to haemorrhagic stroke onset, with a median delay of 15 days in keeping with previous studies [37, 38], also suggesting that a delayed haemorrhagic vasculopathy could be a contributory factor.

In our review, 79% of reported patients were critically ill requiring ICU admission or mechanical ventilation, with very high mortality (54%), consistent with previous studies reporting that ICH was more likely in patients with severe pulmonary COVID-19 (81.8%) with an extremely poor overall prognosis and a mortality rate of 63.6% [39]. Furthermore, 21% of patients received ECMO in our study, which may increase the risk of thrombotic and bleeding complications as indicated by a recent case series that reported 4 out of 10 patients with COVID-19 placed on ECMO developed intracranial haemorrhage, 3 of whom died [40]. By contrast, in non-COVID-19 cohorts, the incidence of ECMO-associated ICH varied between 1.8 and 21% [41].

The incidence of ICH in COVID-19 patients in our study was 0.38% which is lower than the reported incidence of acute ischemic stroke in COVID-19 patients which ranges from 0.9 to 2.7% [42].

The main strength of our study is the inclusion of all available individual patient and aggregate data regarding ICH in patients with COVID-19, allowing us to describe distinct characteristics that are not clearly apparent in small case series. In addition, our review highlights that COVID-19 can be associated with both prothrombotic and haemorrhage-prone states, which might be relevant for anticoagulation strategies.

We acknowledge several limitations of our study. First, we included hospital case series which are likely to be affected by selection and publication bias. Second, we did not have access to data from a contemporaneous control group of patients with non-COVID-19 associated ICH; comparison to historical ICH controls is not likely to be helpful because of the change in the spectrum of stroke presenting to hospital associated with the COVID-19 pandemic [50]. Third, we did not have access to detailed investigations including neuroimaging markers of small vessel disease or vascular imaging to exclude macrovascular causes and assign causes in a systematic and standardised way across cohorts; moreover, limited MRI access during the pandemic might have led to misclassification of haemorrhagic transformation of infarcts as ICHs. Finally, due to the limitations in published data, we were not able to report on the causes of death (for example the proportions attributed to respiratory or neurological disease).

Nevertheless, despite limitations related to the small patient numbers described in most studies, our findings suggest that ICH in COVID-19 has distinct characteristics with potential implications for understanding mechanisms, management of anticoagulation, and clinical trials. Ongoing randomised controlled trials to clarify the risk, benefit, and optimal dose of anticoagulation in patients with COVID-19 should include ICH as a key safety outcome.

## Supplementary Information

Below is the link to the electronic supplementary material.

Supplementary file1 (DOCX 58 KB)
