# Supplementary material for: Characteristics of intracerebral haemorrhage associated with COVID-19: a systematic review and pooled analysis of individual patient and aggregate data
Source: J Neurol. 2021 Feb 5;268(9):3105–15. doi: 10.1007/s00415-021-10425-9 (PMC7864476; doi:10.1007/s00415-021-10425-9)
Supplement: Supplementary file 1 — Supplementary file1 (DOCX 58 KB) [file 415_2021_10425_MOESM1_ESM.docx]

| **Author / Patient** | **Demographic** | **Past Medical History** | **Stroke Characteristics** | **Anticoagulation Treatment** | **Covid Findings** | **Coagulability Markers** |
| --- | --- | --- | --- | --- | --- | --- |
| Heman-Ackah S.M et al.  [1] - Patient 1  - Patient 2 | Age: 58 Sex: Female | Diabetes Systemic lupus erythematosus | Topography: Lobar Focal Mechanism: Coagulopathy | Therapeutic UFH ECMO | Prior Covid Symptoms: Yes Nbr of Days: 25 Covid Severity: Critical Outcome: Deceased | D-Dimer (μg/L): 19,720 Fibrinogen (g/L): 2.5 aPTT (s): 60.4 INR: 1.60 Prothrombin time (s): 18 Platelet count (/ mm3): 188,000 |
|  | Age: 46 Sex: Male | HTN Obstructive sleep apnoea | Topography: Lobar Focal Mechanism: Coagulopathy | Therapeutic UFH ECMO | Prior Covid Symptoms: Yes Nbr of Days: 20 Covid Severity: Critical Outcome: Deceased | D-Dimer (μg/L): 1,040 Fibrinogen (g/L): 6.5 aPTT (s): 70.9 INR: 1.40 Prothrombin time (s): 16 Platelet count (/ mm3): 351,000 |
| Morassi M et al. [2]  - Patient 5  - Patient 6 | Age: 57 Sex: Male | HTN Thrombocytosis | Topography: Lobar Multifocal Mechanism: Coagulopathy | Prophylactic LMWH | Prior Covid Symptoms: Yes Nbr of Days: 18 Covid Severity: Critical Outcome: Deceased | D-Dimer (μg/L): 2,866 aPTT (s): 53.1 CRP (mg/L): 214 |
|  | Age: 57 Sex: Male | No Past Medical History | Topography: Lobar Multifocal Mechanism: Undetermined | Prophylactic LMWH | Prior Covid Symptoms: Yes Nbr of Days: 22 Covid Severity: Critical Outcome: Deceased | CRP (mg/L): 21 |
| Muhammad S et al. [3] - Patient 1 | Age: 60 Sex: Female | N/A | Topography: Lobar Focal Mechanism: aneurysm | No | Prior Covid Symptoms: No Covid Severity: Critical Outcome: Discharged Rehab | CRP (mg/L): 11 |
| Sharifi-Razavi A et al. [4] - Patient 1 | Age: 79 Sex: Male | No History of HTN | Topography: Lobar Focal Mechanism: Undetermined | No | Prior Covid Symptoms: Yes Nbr of Days: 3 Covid Severity: Severe Outcome: Critically Ill | PTT (s): 64.0 INR: 1.00 Prothrombin time (s): 12 Platelet count (/ mm3): 210,000 CRP (mg/L): 10 |
| Vu D et al. [5] - Patient 3 | Age: 30 Sex: Male | No Past Medical History | Topography: Non-lobar Focal Mechanism: Undetermined | No | Prior Covid Symptoms: No Covid Severity: Asymptomatic Outcome: Resolved | Laboratory Variables of Interest N/A |
| Caroll E et al. [6]  - Patient 1  - Patient 2 | Age: 62 Sex: Male | Colon cancer | Topography: Lobar Multifocal Mechanism: Coagulopathy | Therapeutic UFH | Prior Covid Symptoms: Yes Nbr of Days: 20 Covid Severity: Critical Outcome: Deceased | D-Dimer (μg/L): 2,997 Platelet count (/ mm3): 170,000 CRP (mg/L): 165 Serum ferritin (μg/L): 3,072  Supra therapeutic anti-Xa |
|  | Age: 74 Sex: Male | HTN Tongue cancer  Carotid stenosis status post right carotid stent | Topography: Lobar and Non-lobar Multifocal Mechanism: Coagulopathy | Therapeutic UFH | Prior Covid Symptoms: Yes Nbr of Days: 18 Covid Severity: Critical Outcome: Deceased | D-Dimer (μg/L): 1,608 Platelet count (/ mm3): 385,000 CRP (mg/L): 232 Serum ferritin (μg/L): 8,580  Supra therapeutic anti-Xa |
| García-García S et al. [7]  - Patient 1  - Patient 2  - Patient 3  - Patient 4 | Age: 68 Sex: Female | Dyslipidaemia | Topography: Non-lobar Focal Mechanism: Undetermined | No | Prior Covid Symptoms: Yes Nbr of Days: 3 Covid Severity: Moderate Outcome: Resolved | Laboratory Variables of Interest N/A |
|  | Age: 59 Sex: Male | HTN | Topography: Lobar Multifocal Mechanism: Coagulopathy | Therapeutic UFH ECMO | Prior Covid Symptoms: Yes Covid Severity: Critical Outcome: Deceased | Laboratory Variables of Interest N/A |
|  | Age: 60 Sex: Male | N/A | Topography: Lobar Multifocal Mechanism: Coagulopathy | Therapeutic UFH ECMO | Prior Covid Symptoms: Yes Covid Severity: Critical Outcome: Deceased | Laboratory Variables of Interest N/A |
|  | Age: 81 Sex: Female | Diabetes HTN | Topography: Non-lobar Focal Mechanism: Hypertensive | No | Prior Covid Symptoms: N/A Covid Severity: Moderate Outcome: Discharged | Laboratory Variables of Interest N/A |
| Haddadi K et al. [8] - Patient 1 | Age: 54 Sex: Female | Diabetes HTN Lumbar spinal laminectomy and fusion surgery three years ago | Topography: Non-lobar Multifocal Mechanism: Hypertensive | No | Prior Covid Symptoms: Yes Nbr of Days: 5 Covid Severity: Critical Outcome: Resolved | Laboratory Variables of Interest N/A |
| Li J et al. [9] - Patient 1 | Age: 68 Sex: Male | AF | Topography: Lobar Multifocal Mechanism: Coagulopathy | Therapeutic LMWH | Prior Covid Symptoms: Yes Nbr of Days: 19 Covid Severity: Critical Outcome: Deceased | D-Dimer (μg/L): 10,990 INR: 2.06 Prothrombin time (s): 24 |
| Ghosh R et al. [10] - Patient 1 | Age: 19 Sex: Female | No Past Medical History | Topography: Non-lobar Focal Mechanism: Moya Moya angiopathy | No | Prior Covid Symptoms: Yes Nbr of Days: 3 Covid Severity: Mild Outcome: Discharged | Laboratory Variables of Interest N/A |
| Ghani MU et al. [11]  - Patient 1  - Patient 3 | Age: 59 Sex: Male | HTN | Topography: Lobar Focal Mechanism: Coagulopathy | Therapeutic UFH | Prior Covid Symptoms: Yes Nbr of Days: 18 Covid Severity: Critical Outcome: Deceased | D-Dimer (μg/L): 3,000 aPTT (s): 57.0 |
|  | Age: 59 Sex: Female | HTN | Topography: Lobar Focal Mechanism: Coagulopathy | Therapeutic LMWH | Prior Covid Symptoms: Yes Nbr of Days: 15 Covid Severity: Critical Outcome: Deceased | D-Dimer (μg/L): Elevated |
| Wee NK et al. [12] - Patient 1 | Age: 64 Sex: Male | N/A | Topography: Lobar Multifocal Mechanism: Coagulopathy | Therapeutic UFH ECMO | Prior Covid Symptoms: Yes Covid Severity: Critical Outcome: Critically ill | Supratherapeutic aPTT |
| Benger M et al. [13]  - Patient 1  - Patient 2  - Patient 3  - Patient 4  - Patient 5 | Age: 41 Sex: Male | Diabetes HTN | Topography: Lobar Focal Mechanism: Undetermined | Prophylactic LMWH | Prior Covid Symptoms: Yes Nbr of Days: 37 Covid Severity: Critical Outcome: Resolved | D-Dimer (μg/L): 1,920 Fibrinogen (g/L): 3.7  aPTT ratio: 1 INR: 1.00 Platelet count (/ mm3): 510,000 CRP (mg/L): 1 |
|  | Age: 54 Sex: Female | Diabetes HTN DVT/PEs on Warfarin | Topography: Lobar Focal Mechanism: Coagulopathy | Warfarin | Prior Covid Symptoms: Yes Nbr of Days: 14 Covid Severity: Moderate Outcome: Discharged Rehab | D-Dimer (μg/L): 1,400  aPTT ratio: 2.8 INR: 3.20 Platelet count (/ mm3): 270,000 CRP (mg/L): 7 |
|  | Age: 50 Sex: Male | HTN | Topography: Lobar Focal Mechanism: Undetermined | Prophylactic LMWH | Prior Covid Symptoms: Yes Nbr of Days: 32 Covid Severity: Critical Outcome: Critically ill | D-Dimer (μg/L): 8,961  aPTT ratio: 1.3 Fibrinogen (g/L): 7.5 INR: 1.10 Platelet count (/ mm3): 72,000 CRP (mg/L): 138 |
|  | Age: 64 Sex: Female | No Past Medical History | Topography: Non-lobar Focal Mechanism: Coagulopathy | Therapeutic UFH | Prior Covid Symptoms: Yes Nbr of Days: 32 Covid Severity: Critical Outcome: Critically ill | D-Dimer (μg/L): 8,000  aPTT ratio: 2.5 Fibrinogen (g/L): 2.9 INR: 1.10 Platelet count (/ mm3): 221,000 CRP (mg/L): 330 |
|  | Age: 52 Sex: Male | HTN Dyslipidaemia Ischemic HD Quiescent untreated multiple sclerosis, asthma | Topography: Lobar Multifocal Mechanism: Coagulopathy | Therapeutic UFH | Prior Covid Symptoms: Yes Nbr of Days: 38 Covid Severity: Critical Outcome: Critically ill | D-Dimer (μg/L): 7,580  aPTT ratio: 2.9 INR: 1.10 Platelet count (/ mm3): 313,000 CRP (mg/L): 77 |
| D'Anna L et al. [14] - Patient 6 | Age: 55 Sex: Male | HTN Smoking | Topography: Non-lobar Focal Mechanism: Hypertensive | N/A | Prior Covid Symptoms: Yes Nbr of Days: 10 Covid Severity: Moderate Outcome: Discharged | Fibrinogen (g/L): 4.9 aPTT (s): 24.7 Prothrombin time (s): 12 Platelet count (/ mm3): 232,000 CRP (mg/L): 98 |
| Kim C et al. [15] - Patient 1 | Age: 53 Sex: Female | No Past Medical History | Topography: Non-lobar Focal Mechanism: Undetermined | No | Prior Covid Symptoms: No Covid Severity: Asymptomatic Outcome: Discharged Rehab | D-Dimer (μg/L): 410 aPTT (s): 25.6 INR: 1.1. Prothrombin time (s): 11 Platelet count (/ mm3): 141,000 |
| Savic D et al. [16] - Patient 1 | Age: 13 Sex: Female | No Past Medical History | Topography: Lobar Focal Mechanism: pseudoaneurysm | No | Prior Covid Symptoms: No Nbr of Days: 0 Covid Severity: Critical Outcome: Critically Ill | D-Dimer (μg/L): 2,032 CRP (mg/L): 132 |
| Gonçalves B et al. [17]  - Patient 1  - Patient 2  - Patient 3 | Age: 56 Sex: Female | No Past Medical History | Topography: Lobar Multifocal Mechanism: CVT | Prophylactic LMWH | Prior Covid Symptoms: Yes Nbr of Days: 15 Covid Severity: Critical Outcome: Deceased | D-Dimer (μg/L): 59,960 Fibrinogen (g/L): 5.8 Platelet count (/ mm3): 114,000 |
|  | Age: 40 Sex: Male | High BMI | Topography: Lobar and Non-lobar Multifocal Mechanism: Undetermined | Prophylactic LMWH | Prior Covid Symptoms: Yes Nbr of Days: 16 Covid Severity: Critical Outcome: Deceased | D-Dimer (μg/L): 6,746 Fibrinogen (g/L): 4.8 PTT (s): Normal INR: Normal Prothrombin time (s): Normal Platelet count (/ mm3): Normal |
|  | Age: 60 Sex: Female | High BMI  Depression | Topography: Lobar and Non-lobar Multifocal Mechanism: Coagulopathy | Therapeutic UFH | Prior Covid Symptoms: Yes Nbr of Days: 15 Covid Severity: Critical Outcome: Deceased | Fibrinogen (g/L): Normal PTT (s): Normal INR: 2.15 Platelet count (/ mm3): Normal |
| Daci R et al. [18] - Patient 1 | Age: 60 Sex: Female | Asthma  Gastrointestinal reflux  Sleep apnoea | Topography: Non-lobar Multifocal Mechanism: Undetermined | No | Prior Covid Symptoms: Yes Nbr of Days: 2 Covid Severity: Critical Outcome: Deceased | D-Dimer (μg/L): 2,180 Fibrinogen (g/L): 4.4 CRP (mg/L): 124 |
| Zahid M.J et al. [19] - Patient 1 | Age: 38 Sex: Male | No Past Medical History | Topography: Non-lobar Focal Mechanism: Coagulopathy | Therapeutic UFH ECMO | Prior Covid Symptoms: Yes Covid Severity: Critical Outcome: Discharged | Laboratory Variables of Interest N/A |
| Al‑Dalahmah O et al. [20] - Patient 1 | Age: 73 Sex: Male | Diabetes HTN | Topography: Lobar Focal Mechanism: Undetermined | No | Prior Covid Symptoms: No Nbr of Days: 0 Covid Severity: Critical Outcome: Deceased | Fibrinogen (g/L): Normal aPTT (s): Normal PTT (s): Normal INR: Normal Prothrombin time (s): Normal Platelet count (/ mm3): 346,000 CRP (mg/L): 9 |
| Bao Y et al. [21] - Patient 1 | Age: 38 Sex: Male | No Past Medical History | Topography: Lobar Focal Mechanism: Undetermined | No | Prior Covid Symptoms: No Nbr of Days: -1 Covid Severity: Critical Outcome: Critically Ill | CRP (mg/L): 71 |
| Khattare N.L et al. [22] - Patient 1 | Age: 42 Sex: Male | HTN Gastritis | Topography: Lobar Focal Mechanism: Coagulopathy | Therapeutic UFH | Prior Covid Symptoms: Yes Nbr of Days: 29 Covid Severity: Critical Outcome: Deceased | D-Dimer (μg/L): 511 aPTT (s): 66.4 Prothrombin time (s): 10 Platelet count (/ mm3): 200,000 CRP (mg/L): 31 Serum ferritin (μg/L): 409 |
| Usman A.A et al. [23]  - Patient 1  - Patient 2  - Patient 3 | Age: 53.7 Sex: N/A | High BMI | Topography: Lobar Multifocal Mechanism: Coagulopathy | Therapeutic UFH ECMO | Prior Covid Symptoms: N/A Covid Severity: Critical Outcome: Deceased | D-Dimer (μg/L): 19,720 Fibrinogen (g/L): 2.5 aPTT (s): 60.3 Platelet count (/ mm3): 139,000 |
|  | Age: 53.7 Sex: N/A | High BMI | Topography: Lobar Multifocal Mechanism: Coagulopathy | Therapeutic UFH ECMO | Prior Covid Symptoms: N/A Covid Severity: Critical Outcome: Deceased | D-Dimer (μg/L): 1,040 Fibrinogen (g/L): 7.6 aPTT (s): 70.9 Platelet count (/ mm3): 197,000 Serum ferritin (μg/L): 1,596 |
|  | Age: 53.7 Sex: N/A | High BMI | Topography: Lobar Focal Mechanism: Coagulopathy | Therapeutic UFH ECMO | Prior Covid Symptoms: N/A Covid Severity: Critical Outcome: Deceased | D-Dimer (μg/L): 4,850 Fibrinogen (g/L): 5.6 aPTT (s): 37.6 Platelet count (/ mm3): 134,000 Serum ferritin (μg/L): 983 |
| Nicholson P et al. [24]  - Patient 1  - Patient 4 | Age: 43 Sex: Male | N/A | Topography: Lobar Focal Mechanism: Coagulopathy | Therapeutic UFH ECMO | Prior Covid Symptoms: Yes Nbr of Days: 4 Covid Severity: Critical Outcome: Deceased | D-Dimer (μg/L): 4,400 aPTT (s): 63.7 Prothrombin time (s): 13 Platelet count (/ mm3): 172,000 Serum ferritin (μg/L): 2,186 |
|  | Age: 57 Sex: Female | N/A | Topography: Lobar Multifocal Mechanism: Coagulopathy | Therapeutic UFH ECMO | Prior Covid Symptoms: Yes Covid Severity: Critical Outcome: Critically Ill | D-Dimer (μg/L): 4,400 aPTT (s): 48.6 Prothrombin time (s): 12 Platelet count (/ mm3): 127,000 CRP (mg/L): 180 |
| Gogia B et al. [25] - Patient 1 | Age: 75 Sex: Female | HTN Dyslipidaemia Smoking Peripheral artery disease  Hypothyroidism | Topography: Lobar Focal Mechanism: Undetermined | Therapeutic LMWH | Prior Covid Symptoms: Yes Nbr of Days: 17 Covid Severity: Critical Outcome: Deceased | D-Dimer (μg/L): 3,740 Fibrinogen (g/L): 3.9 aPTT (s): 30.0 INR: 1.00 Prothrombin time (s): 12 Platelet count (/ mm3): 201,000 CRP (mg/L): 26 |
| Motoie R et al. [26] - Patient 1 | Age: 50 Sex: Male | Gout | Topography: Lobar Multifocal Mechanism: Coagulopathy | Therapeutic UFH ECMO | Prior Covid Symptoms: Yes Nbr of Days: 10 Covid Severity: Critical Outcome: Deceased | D-Dimer (μg/L): 3,300 Fibrinogen (g/L): 5.1 aPTT (s): 25.8 Prothrombin time (s): 14 Platelet count (/ mm3): 82,000 |
| Fraiman P et al. [27] - Patient 1 | Age: 38 Sex: Female | Alzheimer's disease | Topography: Lobar Focal Mechanism: CAA | No | Prior Covid Symptoms: No Covid Severity: Moderate Outcome: Critically Ill | D-Dimer (μg/L): 3,769 CRP (mg/L): 100 |
| Kvernland A et al. [28]  - Patient 1  - Patient 2  - Patient 3  - Patient 6  - Patient 7  - Patient 8  - Patient 9  - Patient 10  - Patient 11  - Patient 12  - Patient 14  - Patient 15  - Patient 17 | Age: 60 Sex: N/A | Diabetes HTN Dyslipidaemia Mitral and aortic valve replacements on warfarin | Topography: Lobar Focal Mechanism: Coagulopathy | Warfarin | Prior Covid Symptoms: No Nbr of Days: 0 Covid Severity: Critical Outcome: Deceased | D-Dimer (μg/L): 980 Fibrinogen (g/L): 4.6 PTT (s): 43.0 INR: 3.00 Platelet count (/ mm3): 273,000 CRP (mg/L): 11 |
|  | Age: 30 Sex: N/A | Smoking Alcohol | Topography: Lobar Focal Mechanism: Undetermined | No | Prior Covid Symptoms: No Nbr of Days: 0 Covid Severity: Critical Outcome: Critically Ill | D-Dimer (μg/L): 2,138 PTT (s): 35.0 INR: 1.30 Platelet count (/ mm3): 46,000 CRP (mg/L): 56 |
|  | Age: 50 Sex: N/A | AF | Topography: Lobar Focal Mechanism: Coagulopathy | Warfarin | Prior Covid Symptoms: No Nbr of Days: 0 Covid Severity: Critical Outcome: Critically Ill | D-Dimer (μg/L): 231 Fibrinogen (g/L): 3.3 PTT (s): 133.7 INR: 9.00 Platelet count (/ mm3): 77,000 CRP (mg/L): 11 |
|  | Age: 40 Sex: N/A | Diabetes | Topography: Lobar Focal Mechanism: Undetermined | Therapeutic LMWH | Prior Covid Symptoms: Yes Nbr of Days: 35 Covid Severity: Critical Outcome: Discharged Rehab | D-Dimer (μg/L): 506 Fibrinogen (g/L): 5.1 INR: 1,2 Platelet count (/ mm3): 563,000 CRP (mg/L): 143 |
|  | Age: 70 Sex: N/A | HTN Ischemic HD Smoking | Topography: Lobar and Non-lobar Focal Mechanism: Coagulopathy | Therapeutic UFH | Prior Covid Symptoms: Yes Nbr of Days: 18 Covid Severity: Critical Outcome: Deceased | D-Dimer (μg/L): 4,488 Fibrinogen (g/L): 8.9 PTT (s): 77.0 INR: 1.40 Platelet count (/ mm3): 363,000 CRP (mg/L): 416 |
|  | Age: 60 Sex: N/A | Chronic Heart Disease  Active Cancer | Topography: Lobar Focal Mechanism: Coagulopathy | No | Prior Covid Symptoms: Yes Nbr of Days: 2 Covid Severity: Critical Outcome: Deceased | D-Dimer (μg/L): 4,488 PTT (s): 41.9 INR: 1.80 Platelet count (/ mm3): 21,000 CRP (mg/L): 11 |
|  | Age: 50 Sex: N/A | Diabetes HTN Dyslipidaemia | Topography: Lobar Multifocal Mechanism: Coagulopathy | Therapeutic UFH | Prior Covid Symptoms: Yes Nbr of Days: 20 Covid Severity: Critical Outcome: Deceased | D-Dimer (μg/L): 1,791 PTT (s): 41.3 INR: 1.20 Platelet count (/ mm3): 147,000 CRP (mg/L): 137 |
|  | Age: 60 Sex: N/A | HTN Dyslipidaemia Ischemic HD | Topography: Lobar Multifocal Mechanism: Coagulopathy | Therapeutic UFH | Prior Covid Symptoms: Yes Nbr of Days: 26 Covid Severity: Critical Outcome: Deceased | D-Dimer (μg/L): 3,527 Fibrinogen (g/L): 7.1 PTT (s): 61.1 INR: 1.00 Platelet count (/ mm3): 219,000 CRP (mg/L): 44 |
|  | Age: 30 Sex: N/A | Diabetes | Topography: Lobar Multifocal Mechanism: Coagulopathy | Therapeutic UFH | Prior Covid Symptoms: Yes Nbr of Days: 27 Covid Severity: Critical Outcome: Deceased | D-Dimer (μg/L): 5,665 Fibrinogen (g/L): 9.8 PTT (s): 200.0 INR: 6.20 Platelet count (/ mm3): 53,000 CRP (mg/L): 307 |
|  | Age: 60 Sex: N/A | No Past Medical History | Topography: Lobar Multifocal Mechanism: Undetermined | Therapeutic UFH | Prior Covid Symptoms: Yes Nbr of Days: 12 Covid Severity: Critical Outcome: Deceased | D-Dimer (μg/L): 733 Fibrinogen (g/L): 6.5 PTT (s): 80.2 INR: 1.10 Platelet count (/ mm3): 145,000 CRP (mg/L): 61 |
|  | Age: 70 Sex: N/A | No Past Medical History | Topography: Lobar Multifocal Mechanism: Coagulopathy | Therapeutic UFH | Prior Covid Symptoms: Yes Nbr of Days: 16 Covid Severity: Critical Outcome: Critically Ill | D-Dimer (μg/L): 2,622 Fibrinogen (g/L): 7.0 PTT (s): 98.3 INR: 1.26 Platelet count (/ mm3): 228,000 CRP (mg/L): 63 |
|  | Age: 60 Sex: N/A | HIV | Topography: Lobar Focal Mechanism: Cavernous malformation | Therapeutic UFH | Prior Covid Symptoms: Yes Nbr of Days: 17 Covid Severity: Critical Outcome: Discharged home | D-Dimer (μg/L): 3,230 Fibrinogen (g/L): 5.7 PTT (s): 68.8 INR: 1.50 Platelet count (/ mm3): 400,000 CRP (mg/L): 38 |
|  | Age: 60 Sex: N/A | HTN Dyslipidaemia | Topography: Lobar Multifocal Mechanism: Coagulopathy | Therapeutic LMWH | Prior Covid Symptoms: Yes Nbr of Days: 19 Covid Severity: Critical Outcome: Critically Ill | D-Dimer (μg/L): 2,484 Fibrinogen (g/L): 7.0 PTT (s): 27.3 INR: 1.30 Platelet count (/ mm3): 335,000 CRP (mg/L): 105 |
| Agarwal et al. [29]  - Patient 1  - Patient 2 | Age: 56 Sex: Male | HTN Smoking | Topography: Non-lobar Focal Mechanism: Undetermined | No | Prior Covid Symptoms: No Nbr of Days: -1 Covid Severity: Critical Outcome: Critically Ill | INR: 1.07 Platelet count (/ mm3): 220,000 CRP (mg/L): 41 |
|  | Age: 72 Sex: Male | Diabetes | Topography: Lobar Focal Mechanism: Undetermined | No | Prior Covid Symptoms: No Covid Severity: Asymptomatic Outcome: Deceased | INR: 1.00 Platelet count (/ mm3): 165,000 |
| Sai S.T et al. [30] - Patient 1 | Age: 72 Sex: Male | No Past Medical History | Topography: Lobar Focal Mechanism: Undetermined | No | Prior Covid Symptoms: No Covid Severity: Mild Outcome: Discharged | D-Dimer (μg/L): Normal Fibrinogen (g/L): Normal aPTT (s): Normal PTT (s): Normal INR: Normal Prothrombin time (s): Normal Platelet count (/ mm3): Normal CRP (mg/L): 164 |
| Fatehi P et al. [31] - Patient 3 | Age: 50 Sex: Female | No Past Medical History | Topography: Lobar Focal Mechanism: Undetermined | No | Prior Covid Symptoms: No Covid Severity: Critical Outcome: Deceased | Platelet count (/ mm3): 150,000 |
| Castillo P. R et al. [32]  - Patient 1  - Patient 2 | Age: 78 Sex: Female | No Past Medical History | Topography: Non-lobar Focal Mechanism: Undetermined | Prophylactic LMWH | Prior Covid Symptoms: Yes Covid Severity: Critical Outcome: Deceased | D-Dimer (μg/L): Elevated Fibrinogen (g/L): Normal aPTT (s): Normal PTT (s): Normal INR: Normal Prothrombin time (s): Normal Platelet count (/ mm3): Normal CRP (mg/L): Elevated Serum ferritin (μg/L): Elevated |
|  | Age: 65 Sex: Female | HTN | Topography: Lobar Focal Mechanism: Undetermined | Prophylactic LMWH | Prior Covid Symptoms: Yes Covid Severity: Critical Outcome: Deceased | D-Dimer (μg/L): Elevated Fibrinogen (g/L): Normal aPTT (s): Normal PTT (s): Normal INR: Normal Prothrombin time (s): Normal Platelet count (/ mm3): Normal CRP (mg/L): Elevated Serum ferritin (μg/L): Elevated |
| Hernández-Fernández F et al. [33]  - Patient 18  - Patient 19  - Patient 20  - Patient 21  - Patient 22 | Age: 51 Sex: Female | HTN Smoking | Topography: Non-lobar Focal Mechanism: Hypertensive | No | Prior Covid Symptoms: No Covid Severity: Asymptomatic Outcome: Deceased | D-Dimer (μg/L): 3,387 Fibrinogen (g/L): 3.6 Platelet count (/ mm3): 211,000 CRP (mg/L): 110 Serum ferritin (μg/L): 1,554 |
|  | Age: 69 Sex: Male | HTN Dyslipidaemia | Topography: Lobar Focal Mechanism: Coagulopathy | Therapeutic LMWH | Prior Covid Symptoms: Yes Nbr of Days: 13 Covid Severity: Critical Outcome: Critically Ill | D-Dimer (μg/L): 3,387 Fibrinogen (g/L): 3.6 Platelet count (/ mm3): 211,000 CRP (mg/L): 110 Serum ferritin (μg/L): 1,554 |
|  | Age: 68 Sex: Male | No Past Medical History | Topography: Lobar Focal Mechanism: Undetermined | No | Prior Covid Symptoms: No Covid Severity: Critical Outcome: Deceased | D-Dimer (μg/L): 3,387 Fibrinogen (g/L): 3.6 Platelet count (/ mm3): 211,000 CRP (mg/L): 110 Serum ferritin (μg/L): 1,554 |
|  | Age: 64 Sex: Male | Diabetes HTN Dyslipidaemia Stable angina | Topography: Lobar Multifocal Mechanism: Coagulopathy | Therapeutic LMWH | Prior Covid Symptoms: Yes Nbr of Days: 15 Covid Severity: Critical Outcome: Critically Ill | D-Dimer (μg/L): 3,387 Fibrinogen (g/L): 3.6 Platelet count (/ mm3): 211,000 CRP (mg/L): 110 Serum ferritin (μg/L): 1,554 |
|  | Age: 68 Sex: Male | Diabetes HTN Dyslipidaemia Sleep apnoea | Topography: Lobar Focal Mechanism: Coagulopathy | Therapeutic LMWH | Prior Covid Symptoms: Yes Nbr of Days: 16 Covid Severity: Critical Outcome: Critically Ill | D-Dimer (μg/L): 3,387 Fibrinogen (g/L): 3.6 Platelet count (/ mm3): 211,000 CRP (mg/L): 110 Serum ferritin (μg/L): 1,554 |
| Bihlmaier K et al. [34]  - Patient 1  - Patient 2 | Age: 56.6 Sex: Male | Diabetes HTN | Topography: Lobar Multifocal Mechanism: Coagulopathy | Therapeutic LMWH ECMO | Prior Covid Symptoms: Yes Covid Severity: Critical Outcome: Deceased | CRP (mg/L): Elevated Serum ferritin (μg/L): Elevated |
|  | Age: 56.6 Sex: Male | Diabetes HTN | Topography: Lobar Multifocal Mechanism: Coagulopathy | Therapeutic LMWH ECMO | Prior Covid Symptoms: Yes Covid Severity: Critical Outcome: Deceased | CRP (mg/L): Elevated Serum ferritin (μg/L): Elevated |
| D'amore F et al. [35] - Patient 6 | Age: 84 Sex: Female | Diabetes HTN Dyslipidaemia Ovarien carcinoma  Tuberculosis | Topography: Lobar Focal Mechanism: Undetermined | N/A | Prior Covid Symptoms: N/A Nbr of Days: N/A Covid Severity: N/A Outcome: Discharged | Laboratory Variables of Interest N/A |
| Li Y et al. [36] - Patient 11 | Age: 60 Sex: Male | Smoking Alcohol | Topography: Non-lobar Focal Mechanism: Undetermined | N/A | Prior Covid Symptoms: Yes Nbr of Days: 10 Covid Severity: Severe Outcome: Deceased | Laboratory Variables of Interest N/A |
| Shekhar R et al. [37] - Patient 4 | Age: 58 Sex: Female | No Past Medical History | Topography: Lobar Focal Mechanism: Undetermined | Prophylactic LMWH | Prior Covid Symptoms: Yes Nbr of Days: 19 Covid Severity: Critical Outcome: Deceased | D-Dimer (μg/L): 1,425 Platelet count (/ mm3): 239,000 |
| Koh JS et al. [38]  - Patient 1  - Patient 2 | Age: 64 Sex: Male | N/A | Topography: N/A N/A Mechanism: Coagulopathy | Therapeutic UFH ECMO | Prior Covid Symptoms: Yes Nbr of Days: 13 Covid Severity: Critical Outcome: Deceased | aPTT (s): Elevated PTT (s): Elevated |
|  | Age: 60 Sex: Male | N/A | Topography: N/A N/A Mechanism: Coagulopathy | Therapeutic UFH ECMO | Prior Covid Symptoms: Yes Nbr of Days: 21 Covid Severity: Critical Outcome: Deceased | D-Dimer (μg/L): Elevated Fibrinogen (g/L): Elevated aPTT (s): Elevated PTT (s): Elevated |
| Perry R et al. [39]  - Patient 1  - Patient 2  - Patient 3  - Patient 4  - Patient 5 | Age: 76 Sex: Male | No Past Medical History | Topography: Lobar N/A Mechanism: Undetermined | No | Prior Covid Symptoms: No Nbr of Days: -4 Covid Severity: Moderate Outcome: Discharged Home | aPTT (s): 0.0 INR: 1.00 Prothrombin time (s): 12 Platelet count (/ mm3): 273,000 CRP (mg/L): 1 |
|  | Age: 67 Sex: Male | No Past Medical History | Topography: Non-lobar N/A Mechanism: Undetermined | N/A | Prior Covid Symptoms: N/A Covid Severity: Critical Outcome: Deceased | Platelet count (/ mm3): 219,000 CRP (mg/L): 5 |
|  | Age: 50 Sex: Female | HTN | Topography: Non-lobar N/A Mechanism: Hypertensive | No | Prior Covid Symptoms: No Nbr of Days: -2 Covid Severity: Moderate Outcome: Discharged Home | aPTT (s): 22.0 INR: 1.02 Prothrombin time (s): 12 Platelet count (/ mm3): 275,000 CRP (mg/L): 5 |
|  | Age: 76 Sex: Female | HTN | Topography: Non-lobar N/A Mechanism: Hypertensive | No | Prior Covid Symptoms: No Nbr of Days: -4 Covid Severity: Moderate Outcome: Discharged Rehab | aPTT (s): 25.0 Prothrombin time (s): 10 Platelet count (/ mm3): 302,000 CRP (mg/L): 3 |
|  | Age: 79 Sex: Male | HTN | Topography: Non-lobar N/A Mechanism: Hypertensive | N/A | Prior Covid Symptoms: No Covid Severity: Critical Outcome: Discharged Rehab | D-Dimer (μg/L): 744 aPTT (s): 31.0 Prothrombin time (s): 13 Platelet count (/ mm3): 230,000 CRP (mg/L): 2 |
| Hussain A et al. [40]  - Patient 1 | Age: 69 Sex: Female | AF Tissu aortic vavle replacement, Chronic Heart Failure | Topography: Lobar Focal Mechanism: Coagulopathy | Therapeutic LMWH | Prior Covid Symptoms: Yes Nbr of Days: 31 Covid Severity: Critical Outcome: Deceased | Laboratory Variables of Interest N/A |
| Masur J et al. [41]  - Patient 3  - Patient 7  - Patient 9  - Patient 12 | Age: 47 Sex: Male | HTN Obstructive sleep apnea | Topography: Lobar Focal Mechanism: Coagulopathy | Therapeutic UFH ECMO | Prior Covid Symptoms: Yes Covid Severity: Critical Outcome: Deceased | Platelet count (/ mm3): 208,000 |
|  | Age: 59 Sex: Female | Diabetes Systemic lupus erythematosus | Topography: Lobar Multifocal Mechanism: Coagulopathy | Therapeutic UFH ECMO | Prior Covid Symptoms: Yes Covid Severity: Critical Outcome: Deceased | Platelet count (/ mm3): 137,000 |
|  | Age: 60 Sex: Male | HTN Asthma | Topography: Lobar Multifocal Mechanism: Coagulopathy | Therapeutic UFH ECMO | Prior Covid Symptoms: Yes Covid Severity: Critical Outcome: Deceased | Platelet count (/ mm3): 134,000 |
|  | Age: 65 Sex: Female | Diabetes Rheumatoid arthritis | Topography: Lobar Multifocal Mechanism: Coagulopathy | Therapeutic Bivalirudin ECMO | Prior Covid Symptoms: Yes Covid Severity: Critical Outcome: Deceased | Platelet count (/ mm3): 61,000 |
| Urciuoli L et al. [42]  - Patient 2 | Age: 67 Sex: Male | HTN | Topography: Non-lobar Focal Mechanism: Hypertensive | No | Prior Covid Symptoms: Yes Nbr of Days: 7 Covid Severity: Moderate Outcome: Discharged Home | D-Dimer (μg/L): 100 Fibrinogen (g/L): 3.7 aPTT (s): 33.0 Prothrombin time (s): 12 Platelet count (/ mm3): 250,000 |
| Moussa-Ibrahim F et al. [43]  - Patient 1    - Patient 2  - Patient 3  - Patient 4    - Patient 6 | Age: 54 Sex: Female | Diabetes HTN Dyslipidaemia High BMI  Sickle cell trait  Adrenal insufficiency, Hypothyroidism  Asthma  Recurrent supraventricular tachycardia | Topography: Lobar Focal Mechanism: Coagulopathy | Therapeutic UFH | Prior Covid Symptoms: Yes Nbr of Days: 21 Covid Severity: Critical Outcome: Deceased | D-Dimer (μg/L): 29,396 aPTT (s): 25.0 INR: 1.50 Prothrombin time (s): 17 Platelet count (/ mm3): 324,000 CRP (mg/L): 107 Serum ferritin (μg/L): 800 |
|  | Age: 68 Sex: Female | HTN | Topography: Lobar Focal Mechanism: Undetermined | Prophylactic LMWH | Prior Covid Symptoms: Yes Covid Severity: Asymptomatic Outcome: Discharged Rehab | aPTT (s): 28.9 INR: 1.08 Platelet count (/ mm3): 232,000 |
|  | Age: 76 Sex: Male | HTN Dyslipidaemia High BMI  Gout  Gastroesophageal reflux disease | Topography: Lobar Multifocal Mechanism: Coagulopathy | Therapeutic UFH | Prior Covid Symptoms: Yes Nbr of Days: 14 Covid Severity: Critical Outcome: Deceased | D-Dimer (μg/L): 16,107 aPTT (s): 107.0 INR: 1.40 Prothrombin time (s): 16 Platelet count (/ mm3): 161,000 CRP (mg/L): 23 Serum ferritin (μg/L): 3,901 |
|  | Age: 71 Sex: Female | Diabetes HTN Dyslipidaemia High BMI | Topography: Lobar Focal Mechanism: Undetermined | Prophylactic LMWH | Prior Covid Symptoms: Yes Nbr of Days: 26 Covid Severity: Critical Outcome: Deceased | D-Dimer (μg/L): 5,684 aPTT (s): 38.9 INR: 1.10 Prothrombin time (s): 13 Platelet count (/ mm3): 376,000 CRP (mg/L): 19 Serum ferritin (μg/L): 967 |
|  | Age: 63 Sex: Male | Diabetes HTN Smoking | Topography: Lobar Multifocal Mechanism: Coagulopathy | Therapeutic UFH  Thrombolytic therapy using tissue plasminogen activator (occlusive thrombi in the right common femoral artery) | Prior Covid Symptoms: Yes Nbr of Days: 18 Covid Severity: Critical Outcome: Deceased | D-Dimer (μg/L): 13,874 aPTT (s): 30.4 INR: 1.10 Prothrombin time (s): 14 Platelet count (/ mm3): 309,000 CRP (mg/L): 26 Serum ferritin (μg/L): 2,289 |
| Flores G et al. [44]  - Patient 1 | Age: 40 Sex: Male | Diabetes HTN High BMI | Topography: Non-lobar Focal Mechanism: Hypertensive | No | Prior Covid Symptoms: Yes Covid Severity: Critical Outcome: Deceased | D-Dimer (μg/L): Elevated |
| Lopez A.A et al. [45]  - Patient 1  - Patient 2  - Patient 3 | Age: 65 Sex: Male | Diabetes HTN | Topography: Lobar Focal Mechanism: Coagulopathy | Therapeutic LMWH | Prior Covid Symptoms: Yes Nbr of Days: 10 Covid Severity: Critical Outcome: Resolved | D-Dimer (μg/L): 1,600 Platelet count (/ mm3): 95,000 |
|  | Age: 64 Sex: Male | Diabetes HTN | Topography: Lobar Multifocal Mechanism: Coagulopathy | Therapeutic LMWH | Prior Covid Symptoms: Yes Nbr of Days: 12 Covid Severity: Critical Outcome: Resolved | D-Dimer (μg/L): 3,900 Platelet count (/ mm3): 85,000 |
|  | Age: 69 Sex: Male |  | Topography: Lobar Focal Mechanism: Coagulopathy | Therapeutic LMWH | Prior Covid Symptoms: Yes Nbr of Days: 13 Covid Severity: Critical Outcome: Discharged | D-Dimer (μg/L): 7,000 Platelet count (/ mm3): Normal |
| Pavlov V et al. [46]  - Patient 1  - Patient 2  - Patient 3 | Age: 56 Sex: Male | HTN Smoking | Topography: Lobar Focal Mechanism: Undetermined | Prophylactic LMWH | Prior Covid Symptoms: Yes Nbr of Days: 21 Covid Severity: Severe Outcome: Discharged Rehab | D-Dimer (μg/L): 1,820 Fibrinogen (g/L): 2.8 aPTT (s): 22.0 INR: 1.00 Platelet count (/ mm3): 220,000 CRP (mg/L): 88 |
|  | Age: 64 Sex: Male | Diabetes HTN Smoking | Topography: Non-lobar Focal Mechanism: Hypertensive | N/A | Prior Covid Symptoms: Yes Nbr of Days: 10 Covid Severity: Severe Outcome: Discharged Rehab | D-Dimer (μg/L): 2,580 Fibrinogen (g/L): 3.6 aPTT (s): 25.0 INR: 1.10 Platelet count (/ mm3): 334,000 CRP (mg/L): 120 |
|  | Age: 60 Sex: Male | Diabetes HTN Dyslipidaemia | Topography: Non-lobar Focal Mechanism: Hypertensive | N/A | Prior Covid Symptoms: Yes Nbr of Days: 12 Covid Severity: Critical Outcome: Critically Ill | D-Dimer (μg/L): 4,000 Fibrinogen (g/L): 6.5 aPTT (s): 21.0 INR: 1.10 Platelet count (/ mm3): 480,000 CRP (mg/L): 189 |
| Fayed I et al. [47]  - Patient 1 | Age: 57 Sex: Female | HTN High BMI, Asthma, Sleep apnoea | Topography: Lobar Focal Mechanism: Undetermined | Therapeutic UFH | Prior Covid Symptoms: Yes Nbr of Days: 20 Covid Severity: Critical Outcome: Discharged Rehab | D-Dimer (μg/L): 14,080 Fibrinogen (g/L): 6.4 aPTT (s): 40.9 INR: 1.20 Prothrombin time (s): 16 Platelet count (/ mm3): 506,000 |

AF-atrial fibrillation; BMI-body mass index; CRP-C-reactive protein; ECMO-extracorporeal membrane oxygenation; HTN-hypertension; INR-international normalised ratio; LMWH-low molecular weight heparin; aPTT-activated partial thromboplastin time; PTT-prothrombin time; UFH-unfractionated heparin

**References**

1. Heman-Ackah S M, Su Y S, Spadola M, Petrov D, Chen H I, et al Neurologically Devastating Intraparenchymal Hemorrhage in COVID-19 Patients on Extracorporeal Membrane Oxygenation: A Case Series. Neurosurg. 2020; 87 (2): E147–E151, <https://doi.org/10.1093/neuros/nyaa198>
2. Morassi M, Bagatto D, Cobelli M, et al. Stroke in patients with SARS-CoV-2 infection: case series. J Neurol. 2020;267(8):2185-2192. doi:10.1007/s00415-020-09885-2
3. Muhammad S, Petridis A, Cornelius JF, Hänggi D. Letter to editor: Severe brain haemorrhage and concomitant COVID-19 Infection: A neurovascular complication of COVID-19. Brain Behav Immun. 2020;87:150-151. doi:10.1016/j.bbi.2020.05.015
4. Sharifi-Razavi A, Karimi N, Rouhani N. COVID-19 and intracerebral haemorrhage: causative or coincidental? New Microbes New Infect. 2020 Mar 27;35:100669. doi: 10.1016/j.nmni.2020.100669. PMID: 32322398; PMCID: PMC7163302.
5. Vu D, Ruggiero M, Choi WS, et al. Three unsuspected CT diagnoses of COVID-19. Emerg Radiol. 2020;27(3):229-232. doi:10.1007/s10140-020-01775-4
6. Carroll E, Lewis A. Catastrophic Intracranial Hemorrhage in Two Critically Ill Patients with COVID-19 [published online ahead of print, 2020 May 26]. Neurocrit Care. 2020;1-5. doi:10.1007/s12028-020-00993-5
7. García-García S, Cepeda S, Arrese I, Sarabia R. Letter: Hemorrhagic Conditions Affecting the Central Nervous System in COVID-19 Patients. Neurosurgery. 2020 Sep 1;87(3):E394-E396. doi: 10.1093/neuros/nyaa253. PMID: 32490520; PMCID: PMC7313785.
8. Haddadi K, Ghasemian R, Shafizad M. Basal Ganglia Involvement and Altered Mental Status: A Unique Neurological Manifestation of Coronavirus Disease 2019. Cureus. 2020;12(4):e7869. Published 2020 Apr 28. doi:10.7759/cureus.7869
9. Li J, Long X, Zhu C, et al. A case of COVID-19 pneumonia with cerebral hemorrhage. Thromb Res. 2020;193:22-24. doi:10.1016/j.thromres.2020.05.050
10. Ghosh R, Dubey S, Kanti Ray B, Chatterjee S, Benito-León J. COVID-19 Presenting With Thalamic Hemorrhage Unmasking Moyamoya Angiopathy [published online ahead of print, 2020 Jun 4]. Can J Neurol Sci. 2020;1-3. doi:10.1017/cjn.2020.117
11. Ghani MU, Kumar M, Ghani U, Sonia F, Abbas SA. Intracranial hemorrhage complicating anticoagulant prophylactic therapy in three hospitalized COVID-19 patients. J Neurovirol. 2020;26(4):602-604. doi:10.1007/s13365-020-00869-6
12. Wee NK, Fan EB, Lee KCH, Chia YW, Lim TCC. CT Fluid-Blood Levels in COVID-19 Intracranial Hemorrhage. AJNR Am J Neuroradiol. 2020 Sep;41(9):E76-E77. doi: 10.3174/ajnr.A6672. Epub 2020 Jun 18. PMID: 32554426.
13. Benger M, Williams O, Siddiqui J, Sztriha L. Intracerebral haemorrhage and COVID-19: Clinical characteristics from a case series. Brain Behav Immun. 2020;88:940-944. doi:10.1016/j.bbi.2020.06.005
14. D'Anna L, Kwan J, Brown Z, et al. Characteristics and clinical course of Covid-19 patients admitted with acute stroke [published online ahead of print, 2020 Jun 24]. J Neurol. 2020;1-5. doi:10.1007/s00415-020-10012-4
15. Kim C, Kwak Y, Hwang J, Eun MY. Spontaneous Intracerebral Hemorrhage in a Patient with Asymptomatic 2019 Novel Coronavirus Disease. J Clin Neurol. 2020;16(3):515-517. doi:10.3988/jcn.2020.16.3.515
16. Savić D, Alsheikh TM, Alhaj AK, et al. Ruptured cerebral pseudoaneurysm in an adolescent as an early onset of COVID-19 infection: case report. Acta Neurochir (Wien). 2020;162(11):2725-2729. doi:10.1007/s00701-020-04510-7
17. Gonçalves B, Righy C, Kurtz P. Thrombotic and Hemorrhagic Neurological Complications in Critically Ill COVID-19 Patients. Neurocrit Care. 2020 Oct;33(2):587-590. doi: 10.1007/s12028-020-01078-z. Epub 2020 Aug 12. PMID: 32789603.
18. Daci R, Kennelly M, Ferris A, Azeem MU, Johnson MD, Hamzei-Sichani F, Jun-O'Connell AH, Natarajan SK. Bilateral Basal Ganglia Hemorrhage in a Patient with Confirmed COVID-19. AJNR Am J Neuroradiol. 2020 Oct;41(10):1797-1799. doi: 10.3174/ajnr.A6712. Epub 2020 Aug 20. PMID: 32819902.
19. Zahid MJ, Baig A, Galvez-Jimenez N, Martinez N. Hemorrhagic stroke in setting of severe COVID-19 infection requiring Extracorporeal Membrane Oxygenation (ECMO). J Stroke Cerebrovasc Dis. 2020 Sep;29(9):105016. doi: 10.1016/j.jstrokecerebrovasdis.2020.105016. Epub 2020 Jun 6. PMID: 32807431; PMCID: PMC7275181.
20. Al-Dalahmah, O., Thakur, K.T., Nordvig, A.S. et al. Neuronophagia and microglial nodules in a SARS-CoV-2 patient with cerebellar hemorrhage. acta neuropathol commun 8, 147 (2020). <https://doi.org/10.1186/s40478-020-01024-2>
21. Bao Y, Lin SY, Cheng ZH, et al. Clinical Features of COVID-19 in a Young Man with Massive Cerebral Hemorrhage-Case Report [published online ahead of print, 2020 May 23]. SN Compr Clin Med. 2020;1-7. doi:10.1007/s42399-020-00315-y
22. Khattar, N. K., Sharma, M., Mccallum, A. P., &amp; Oxford, B. G. (2020). Intracranial hemorrhage in a young COVID-19 patient. Interdisciplinary Neurosurgery, 22, 100878. doi:10.1016/j.inat.2020.100878
23. Usman AA, Han J, Acker A, et al. A Case Series of Devastating Intracranial Hemorrhage During Venovenous Extracorporeal Membrane Oxygenation for COVID-19. J Cardiothorac Vasc Anesth. 2020;34(11):3006-3012. doi:10.1053/j.jvca.2020.07.063
24. Nicholson P, Alshafai L, Krings T. Neuroimaging Findings in Patients with COVID-19. AJNR Am J Neuroradiol. 2020 Aug;41(8):1380-1383. doi: 10.3174/ajnr.A6630. Epub 2020 Jun 11. PMID: 32527843.
25. Gogia B, Fang X, Rai P. Intracranial Hemorrhage in a Patient With COVID-19: Possible Explanations and Considerations. Cureus. 2020;12(8):e10159. Published 2020 Aug 31. doi:10.7759/cureus.10159
26. Motoie R, Akai M, Kitahara T, Imamura H, Tanabe T, Sarazawa K, Takano S, Toda H, Komatsu K. Coronavirus Disease 2019 Complicated by Multiple Simultaneous Intracerebral Hemorrhages. Intern Med. 2020 Sep 5. doi: 10.2169/internalmedicine.5697-20. Epub ahead of print. PMID: 32893231.
27. Fraiman, P., Freire, M., Moreira-Neto, M., &amp; Godeiro-Junior, C. (2020). Hemorrhagic stroke and COVID-19 infection: Coincidence or causality? ENeurologicalSci, 21, 100274. doi:10.1016/j.ensci.2020.100274
28. Kvernland A, Kumar A, Yaghi S, et al. Anticoagulation use and Hemorrhagic Stroke in SARS-CoV-2 Patients Treated at a New York Healthcare System [published online ahead of print, 2020 Aug 24]. Neurocrit Care. 2020;1-12. doi:10.1007/s12028-020-01077-0
29. Agarwal A, Vishnu VY, Vibha D, et al. Intracerebral Hemorrhage and SARS-CoV-2: Association or Causation. Ann Indian Acad Neurol. 2020;23(3):261-264. doi:10.4103/aian.AIAN_362_20
30. Thu SS, Matin N, Levine SR. Olfactory gyrus intracerebral hemorrhage in a patient with COVID-19 infection. J Clin Neurosci. 2020;79:275-276. doi:10.1016/j.jocn.2020.07.033
31. Fatehi P, Hesam-Shariati N, Abouzaripour M, Fathi F, Hesam Shariati MB. Acute Ischemic and Hemorrhagic Stroke and COVID-19: Case Series [published online ahead of print, 2020 Oct 1]. SN Compr Clin Med. 2020;1-6. doi:10.1007/s42399-020-00559-8
32. Castillo PR, Del Brutto OH, Mautong H, et al. Hemorrhagic stroke in hispanics with severe SARS-CoV2 infection [published online ahead of print, 2020 Jul 16]. J Neurol Sci. 2020;416:117046. doi:10.1016/j.jns.2020.117046
33. Hernández-Fernández F, Valencia HS, Barbella-Aponte RA, et al. Cerebrovascular disease in patients with COVID-19: neuroimaging, histological and clinical description [published online ahead of print, 2020 Jul 9]. Brain. 2020;awaa239. doi:10.1093/brain/awaa239
34. Bihlmaier K, Coras R, Willam C, et al. Disseminated Multifocal Intracerebral Bleeding Events in Three Coronavirus Disease 2019 Patients on Extracorporeal Membrane Oxygenation As Rescue Therapy. Crit Care Explor. 2020;2(9):e0218. Published 2020 Sep 15. doi:10.1097/CCE.0000000000000218
35. D'Amore F, Vinacci G, Agosti E, Cariddi LP, Terrana AV, Vizzari FA, Mauri M, Giorgianni A. Pressing Issues in COVID-19: Probable Cause to Seize SARS-CoV-2 for Its Preferential Involvement of Posterior Circulation Manifesting as Severe Posterior Reversible Encephalopathy Syndrome and Posterior Strokes. AJNR Am J Neuroradiol. 2020 Oct;41(10):1800-1803. doi: 10.3174/ajnr.A6679. Epub 2020 Jul 30. PMID: 32732268.
36. Li Y, Li M, Wang M, Zhou Y, Chang J, Xian Y, Wang D, Mao L, Jin H, Hu B. Acute cerebrovascular disease following COVID-19: a single center, retrospective, observational study. Stroke Vasc Neurol. 2020 Sep;5(3):279-284. doi: 10.1136/svn-2020-000431. Epub 2020 Jul 2. PMID: 32616524; PMCID: PMC7371480.
37. Shekhar R, Sheikh AB, Suriya SS, Upadhyay S, Zafar A. Neurological Complications Among Native Americans with COVID-19: Our Experience at a Tertiary Care Academic Hospital in the U.S [published online ahead of print, 2020 Aug 24]. J Stroke Cerebrovasc Dis. 2020;29(12):105260. doi:10.1016/j.jstrokecerebrovasdis.2020.105260
38. Koh JS, De Silva DA, Quek AML, Chiew HJ, Tu TM, Seet CYH, Hoe RHM, Saini M, Hui AC, Angon J, Ker JR, Yong MH, Goh Y, Yu WY, Lim TCC, Tan BYQ, Ng KWP, Yeo LLL, Pang YZ, Prakash KM, Ahmad A, Thomas T, Lye DCB, Tan K, Umapathi T. Neurology of COVID-19 in Singapore. J Neurol Sci. 2020 Sep 3;418:117118. doi: 10.1016/j.jns.2020.117118. Epub ahead of print. PMID: 32977228; PMCID: PMC7470792.
39. Perry R J, Smith C J, Roffe C, Simister R Jet al. Characteristics and outcomes of COVID-19-associated stroke: a UK multicentre case-control study Journal of Neurology, Neurosurgery & Psychiatry 2020; “article in press”; <http://dx.doi.org/10.1136/jnnp-2020-324927>
40. Hussain A, Roberts N, Oo A. Prosthetic aortic valve endocarditis complicated by COVID-19 and hemorrhage. J Card Surg. 2020;35(6):1348-1350. doi:10.1111/jocs.14643
41. Masur J, Freeman CW, Mohan S. A Double-Edged Sword: Neurologic Complications and Mortality in Extracorporeal Membrane Oxygenation Therapy for COVID-19-Related Severe Acute Respiratory Distress Syndrome at a Tertiary Care Center. AJNR Am J Neuroradiol. 2020 Aug 27. doi: 10.3174/ajnr.A6728. Epub ahead of print. PMID: 32855187.
42. Urciuoli L, Guerriero E, Musto L. ACUTE ISCHEMIC AND HEMORRHAGIC STROKE IN TWO COVID-19 PATIENTS. Jpn J Infect Dis. 2020 Oct 30. doi: 10.7883/yoken.JJID.2020.448. Epub ahead of print. PMID: 33132298.
43. Mousa-Ibrahim F, Berg S, Odetola O, Teitcher M, Ruland S. Intracranial Hemorrhage in Hospitalized SARS-CoV-2 Patients: A Case Series [published online ahead of print, 2020 Nov 2]. J Stroke Cerebrovasc Dis. 2020;30(1):105428. doi:10.1016/j.jstrokecerebrovasdis.2020.105428
44. Flores G, Kumar JI, Pressman E, Sack J, Alikhani P. Spontaneous Brainstem Hemorrhagic Stroke in the Setting of Novel Coronavirus Disease 2019 - A Case Report. Cureus. 2020;12(10):e10809. Published 2020 Oct 5. doi:10.7759/cureus.10809
45. Garví López M, Tauler Redondo MDP, Tortajada Soler JJ. Intracranial hemorrhages in critical COVID-19 patients: report of three cases [published online ahead of print, 2020 Sep 25]. Intracranial hemorrhages in critical COVID-19 patients: report of three cases [published online ahead of print, 2020 Sep 25]. Med Clin (Barc) . 2020; S0025-7753 (20) 30667-9. doi: 10.1016 / j.medcli.2020.08.005
46. Pavlov V, Beylerli O, Gareev I, Torres Solis LF, Solís Herrera A, Aliev G. COVID-19-Related Intracerebral Hemorrhage. Front Aging Neurosci. 2020 Oct 22;12:600172. doi: 10.3389/fnagi.2020.600172. PMID: 33192492; PMCID: PMC7642875.
47. Fayed I, Pivazyan G, Conte AG, Chang J, Mai JC. Intracranial hemorrhage in critically ill patients hospitalized for COVID-19. J Clin Neurosci. 2020;81:192-195. doi:10.1016/j.jocn.2020.08.026
